# Colorless Iodine

**Published:** 1866-02

**Authors:** 


### "COLORLESS IODINE."

Dentists often feel that the local action of Iodine is desirable, where its use is interdicted on account of the unsightly color it imparts to the cuticle. In addition to the color imparted, tincture of iodine forms a sort of incrustation with the cuticle, that retards the absorption of subsequent applications. Both of these objections may be obviated as follows:

Take of

Tincturæ Iodini Comp.

Aquæ Ammoniæ fort. aa ℥j.

Camphoræ ℈j.

mix.

Let it stand till transparent. This is rapidly absorbed by the skin without change of color. The camphor may be omitted, if its action is not desired. In the treatment of the antrum this is a good external lotion. We obtained the formula from a medical friend, and are not able to give the proper credit for its discovery. W.

